# Gpr17 deficiency in POMC neurons ameliorates the metabolic derangements caused by long-term high-fat diet feeding

**DOI:** 10.1038/s41387-019-0096-7

**Published:** 2019-10-14

**Authors:** Austin M. Reilly, Shudi Zhou, Sunil K. Panigrahi, Shijun Yan, Jason M. Conley, Patrick L. Sheets, Sharon L. Wardlaw, Hongxia Ren

**Affiliations:** 1 Stark Neurosciences Research Institute, Medical Neuroscience Graduate Program, Indiana University School of Medicine, 320 W. 15th Street Indianapolis, Indianapolis, IN 46202 USA; 2 Department of Medicine, Division of Endocrinology, Vagelos College of Physicians and Surgeons, Columbia University, New York, NY 10032 USA; 3 Herman B. Wells Center for Pediatric Research, Department of Pediatrics, Indiana University School of Medicine, 635 Barnhill Drive, Indianapolis, IN 46202 USA; 4 Center for Diabetes and Metabolic Diseases, Indiana University School of Medicine, 635 Barnhill Drive, Indianapolis, IN 46202 USA; 5 Department of Pharmacology & Toxicology, Indiana University School of Medicine, 635 Barnhill Drive, Indianapolis, IN 46202 USA; 6 Department of Cellular & Integrative Physiology, Indiana University School of Medicine, 635 Barnhill Drive, Indianapolis, IN 46202 USA; 7 Department of Biochemistry & Molecular Biology, Indiana University School of Medicine, 635 Barnhill Drive, Indianapolis, IN 46202 USA

**Keywords:** Homeostasis, Ageing, Feeding behaviour

## Abstract

**Background:**

Proopiomelanocortin (POMC) neurons in the arcuate nucleus of the hypothalamus (ARH) control energy homeostasis by sensing hormonal and nutrient cues and activating secondary melanocortin sensing neurons. We identified the expression of a G protein-coupled receptor, Gpr17, in the ARH and hypothesized that it contributes to the regulatory function of POMC neurons on metabolism.

**Methods:**

In order to test this hypothesis, we generated POMC neuron-specific *Gpr17* knockout (PGKO) mice and determined their energy and glucose metabolic phenotypes on normal chow diet (NCD) and high-fat diet (HFD).

**Results:**

Adult PGKO mice on NCD displayed comparable body composition and metabolic features measured by indirect calorimetry. By contrast, PGKO mice on HFD demonstrated a sexually dimorphic phenotype with female PGKO mice displaying better metabolic homeostasis. Notably, female PGKO mice gained significantly less body weight and adiposity (*p* < 0.01), which was associated with increased energy expenditure, locomotor activity, and respiratory quotient, while males did not have an overt change in energy homeostasis. Though PGKO mice of both sexes had comparable glucose and insulin tolerance, detailed analyses of liver gene expression and serum metabolites indicate that PGKO mice could have reduced gluconeogenesis and increased lipid utilization on HFD. To elucidate the central-based mechanism(s) underlying the better-preserved energy and glucose homeostasis in PGKO mice on HFD, we examined the electrophysiological properties of POMC neurons and found Gpr17 deficiency led to increased spontaneous action potentials. Moreover, PGKO mice, especially female knockouts, had increased POMC-derived alpha-melanocyte stimulating hormone and beta-endorphin despite a comparable level of prohormone POMC in their hypothalamic extracts.

**Conclusions:**

Gpr17 deficiency in POMC neurons protects metabolic homeostasis in a sex-dependent manner during dietary and aging challenges, suggesting that Gpr17 could be an effective anti-obesity target in specific populations with poor metabolic control.

## Introduction

Chronic food intake in excess of energy expenditure leads to adiposity gain and obesity. Proopiomelanocortin (POMC) neurons in the arcuate nucleus of the hypothalamus (ARH) control energy homeostasis through the production of alpha-melanocyte stimulating hormone (α-MSH), a POMC-derived peptide which activates melanocortin receptors 3 and 4 (MC3/4R)^1–5^. Human patients with loss-of-function mutations in *POMC* or *MC3/4R* have persistent hunger and consequently develop obesity^5,6^, highlighting the crucial role of melanocortin signaling in managing energy balance. Neurons co-expressing Agouti-related peptide and Neuropeptide Y (AgRP/NPY) in the ARH decrease satiety by opposing the functions of POMC neurons via GABAergic projections onto POMC neurons and the secretion of AgRP and NPY neuropeptides^7–12^. POMC neurons and AgRP/NPY neurons have divergent responses to adiposity signals^13–15^. Given the pivotal role of POMC neurons in regulating metabolic homeostasis, our research objective was to identify novel mechanisms controlling POMC neuronal activity that can be leveraged for treating obesity.

Although the role of POMC neurons in managing energy balance is well established, the biological mechanisms regulating their activity is still an area under active investigation. Forkhead box protein O1 (FoxO1) protein was detected in the hypothalamic AgRP and POMC neurons, and hypothalamic expression of a constitutive active form of FoxO1 resulted in a loss of the ability of leptin to curtail food intake^16^. Carboxypeptidase E (Cpe), an enzyme that mediates POMC processing, was identified as a FoxO1 transcriptional target in POMC neurons^17^. We, as well as other groups, identified Gpr17 as a transcriptional target of FoxO1 in the central nervous system^18,19^. Furthermore, we generated Gpr17 conditional knockout mice and analyzed its metabolic function in AgRP neurons^18^.

Based on emerging evidence that the orphan receptor Gpr17 is expressed by neuronal populations involved in energy homeostasis^18,20^, we hypothesized that Gpr17 signaling regulates POMC neuronal function to control appetite, metabolism, and energy homeostasis. In order to test this hypothesis, we generated POMC neuron-specific Gpr17 knockout mice and determined their basal metabolic features. Gender differences exist in regulation of metabolism^21^. POMC neurons exhibit sexual dimorphism in the regulation of energy homeostasis^22,23^. Moreover, aging and unhealthy diet are known factors associated with adiposity gain, a major contributor to insulin resistance and metabolic derangements. Therefore, in this study, we analyzed the metabolic phenotype of both female and male mice at different ages challenged with chronic feeding of high-fat diet. Our systemic characterization of the mutant mice of both sexes revealed that Gpr17 deficiency in POMC neurons ameliorated the metabolic derangements caused by long-term high-fat diet feeding, which was more pronounced in female mice.

## Materials and methods

### Experimental animals

*Pomc* promoter-driven *Gpr17* knockout (PGKO) mice were generated by cross-breeding *Pomc-Cre* mice^24^ and *Gpr17 lox/lox* mice^20^. *Pomc-Cre* negative, *Gpr17 lox/lox* mice, or *Pomc-Cre;Gpr17 lox/+*mice were used as controls for all experiments. All mice were maintained in the Columbia University or IUSM Lab animal resource center (LARC) facility. The procedures were approved by the Columbia University Animal Care and Utilization Committee and Indiana University Animal Care and Use Committee (IACUC). Mice were weaned at 3 weeks old and group housed (3–5 mice per cage) except during indirect calorimetry measurements (1 mouse per cage). Animals were assigned for experiments blinded and genetic information was revealed only after experiment was completed.

### Feeding regimen

Experimental animals were fed with either normal chow diet (NCD) or high-fat diet (HFD). NCD had 62.1% of calories from carbohydrates, 24.6% from protein, and 13.2% from fat (PicoLab Rodent Diet 20, catalog #5053; Purina Mills. In order to induce obesity, we used high-fat diet (HFD) containing 60% calories from fat, 20% from protein, 20% from carbohydrate (Research Diets, catalog #D12492, New Brunswick, NJ). We profiled the metabolic phenotype in adult cohorts fed NCD, HFD for 4–5 months and for 8–10 months (i.e., long-term HFD) as indicated by the figure legends.

### Serum biochemistries

Mouse serum was collected by tail vein bleeding or terminal cardiac puncture. In order to characterize the metabolic flexibility of mice, we analyzed serum metabolite concentrations during ad libitum feeding, short fasting during daytime (5 h), overnight fasting (~16 h), and refeeding (4–5 h of ad libitum feeding after overnight fasting). We measured free glycerol (Sigma cat. no. F6428), free cholesterol (Wako cat. no. 993-02501), and non-esterified free fatty acid (Wako cat. nos. 999-34691, 995-34791, 991-34891, 993-35191) by colorimetric enzymatic assays. We used ELISAs to measure serum leptin (EMD Millipore cat. no. EZML-82K), and serum insulin (EMD Millipore cat. no. EZRMI-13K). All reactions were performed according to manufacturer protocols.

### Indirect calorimetry

Indirect calorimetry measurements (food intake, energy expenditure, respiratory quotient, and locomotor activity) were collected using a TSE PhenoMaster Platform (TSE Systems, Chesterfield, MO) as described previously^25^, except for the female long-term HFD cohort. Briefly, mice were single-housed for the duration of metabolic analysis and were allowed to acclimate for 48 h before analysis. Data were collected every 51 min during a normal 12-h day/night cycle. Mice had ad libitum access to normal or high-fat diet chow. Total body weight and lean mass were determined beforehand by MRI scan (EcoMRI-100, EcoMRI Houston, TX) for calculating indirect calorimetry measurements. Long-term HFD females were analyzed on Comprehensive Laboratory Animal Monitoring System (CLAMS, Oxymax Windows 3.0.3; Columbus Instruments). CLAMS data were collected in the same manner at 17–30 min intervals during a normal 12-h day/night cycle.

### Quantification of POMC and POMC-derived peptides

PGKO and control mice aged 3–6 months were exposed to HFD for 2 weeks, then sacrificed after ad libitum feeding and peptides were measured in acid extracts of dissected medial basal hypothalamic samples as previously described^26^. POMC was measured by 2-site ELISA with antibodies provided by Dr. Anne White (University of Manchester, UK); there is no cross-reactivity with α-MSH or β-EP^26^. α-MSH and β-EP were measured by radioimmunoassay (RIA)^26^. The β-EP antibody fully cross-reacts with β-EP (1–31), β-EP (1–26), and β-EP (1–27) and 2.6% with POMC on a molar basis. The α-MSH antibody fully cross-reacts with desacetyl α-MSH; there is no cross-reactivity with β-EP, adrenocorticotropin hormone (ACTH), or POMC. The total protein concentration was measured in each hypothalamic sample and used for normalization in order to minimize the variability of individual sample dissection and extraction.

### Glucose tolerance test and insulin tolerance test

Glucose tolerance test (GTT) was performed as described previously^25^ in PGKO and WT mice fed HFD for 4–5 months. Briefly, we injected a bolus of glucose solution intraperitoneally (1 g/kg in males and 2 g/kg in females) after an overnight fast (~16 h). Blood glucose concentrations were measured from drops of tail blood at the indicated time points using a glucose meter (Bayer Breeze2). In order to determine the sensitivity of peripheral tissues to insulin, we performed insulin tolerance tests (ITT) to mice after 6 h of fasting. We delivered 1.5 Units/kg of insulin (Novo Nordisk) by intraperitoneal injection and recorded tail blood glucose concentration over time (0, 15, 30, 60, 90 min).

### Gene expression

Livers and brains were collected from mice euthanized with CO_2_ after feeding as indicated in the corresponding figure legend. We extracted RNA with TRIzol Reagent (Invitrogen) and used Superscript II reverse transcriptase (Invitrogen) to synthesize template cDNA for quantitative RT-PCR. We PCR amplified target DNA with primers spanning introns. Primer sequences are available upon request. All reactions were performed according to manufacturer protocols.

### Statistical comparisons

We used GraphPad Prism software for statistical analyses and graphics generation. Data are presented as average ± standard error. Comparisons were made between age-matched control and knockout littermates. Statistical tests and group sizes are specified in the corresponding Figure legend. Sample exclusion criteria were predetermined. For endpoint experiments, we excluded outliers more than 2 SD from the group mean. Mice with clearly abnormal behavior, such as hyperlocomotion or lack of food intake, were excluded from metabolic analysis.

### Electrophysiology, histology, and fluorescence activated cell sorting (FACS)

Detailed methods are described in ‘Supplemental Methods’.

## Results

### PGKO male and female mice have normal body weight and composition on normal chow diet (NCD)

In order to understand the physiological role of Gpr17 in POMC neurons, we specifically knocked out *Gpr17* in POMC neurons (*Pomc-Cre;Gpr17 lox/lox* mice, hereafter called PGKO mice). PGKO mice were compared with littermate control mice in individual cohorts (hereafter called wild-type (WT) mice).

In order to characterize the specificity and efficiency of Cre-dependent knockout, we first extracted genomic DNA from various tissues and were able to detect the recombined allele in the mediobasal hypothalamus (mbh) but not in other tissues (Fig. S1A, arrow). In order to specifically assess the gene expression of *Gpr17* in POMC neurons in the WT and PGKO mice, we used the fluorescence activated cell sorting (FACS) of live dissociated hypothalamic cells. We introduced a reporter *Rosa-tdTomato* to specifically label POMC neurons in WT and PGKO mice, then collected the Tomato + cell population for gene expression analysis with RT-PCR. *Gpr17* transcript was virtually undetectable in the POMC neurons of the PGKO mice, while it was detected in the POMC neurons of the WT mice as well as the input fractions (Fig. S1B–D). FACS successfully enriched the *Pomc* transcripts (~400 fold) in the Tomato + fraction, which further validated our method (Fig. S1E). Overall, this set of experiments demonstrated the successful and specific ablation of *Gpr17* expression in the POMC neurons of PGKO mice.

**Fig. 1:**
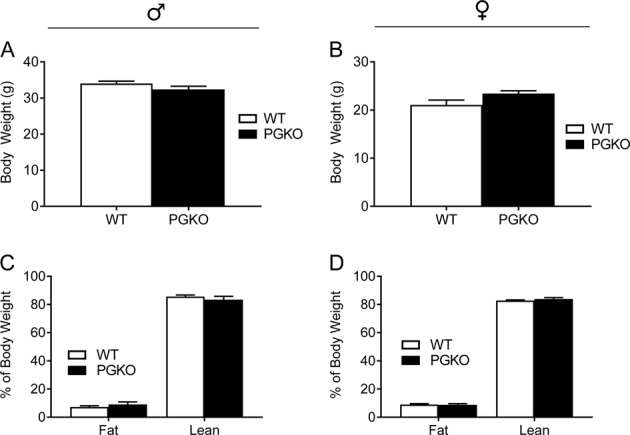
PGKO male and female mice have normal body weight and composition on NCD. Left-hand data are male mice, right-hand data are female mice. Body weight in grams is presented for males (**a**) and females (**b**). Fat and lean mass percentage for male (**c**) and female (**d**) mice were measured with MRI prior to indirect calorimetry. Statistical comparisons with unpaired student’s *t*-test were non-significant (*n* = 8, 7 males; *n* = 9, 7 females). Data shown are average ± standard error

We characterized body composition and energy homeostasis in PGKO mice on normal chow diet. PGKO males and females had comparable total body weight with control males and females, respectively (Fig. 1a, b). We measured body composition with MRI and found that fat mass (percentage of body weight) and lean mass (percentage of body weight) were similar between PGKO and control mice (Fig. 1c, d). Therefore, we concluded that PGKO mice on a normal chow diet were able to maintain a healthy body weight and composition similarly to control mice without an apparent defect in growth or energy balance.

### Feeding and energy balance in NCD-fed PGKO mice

**Fig. 2:**
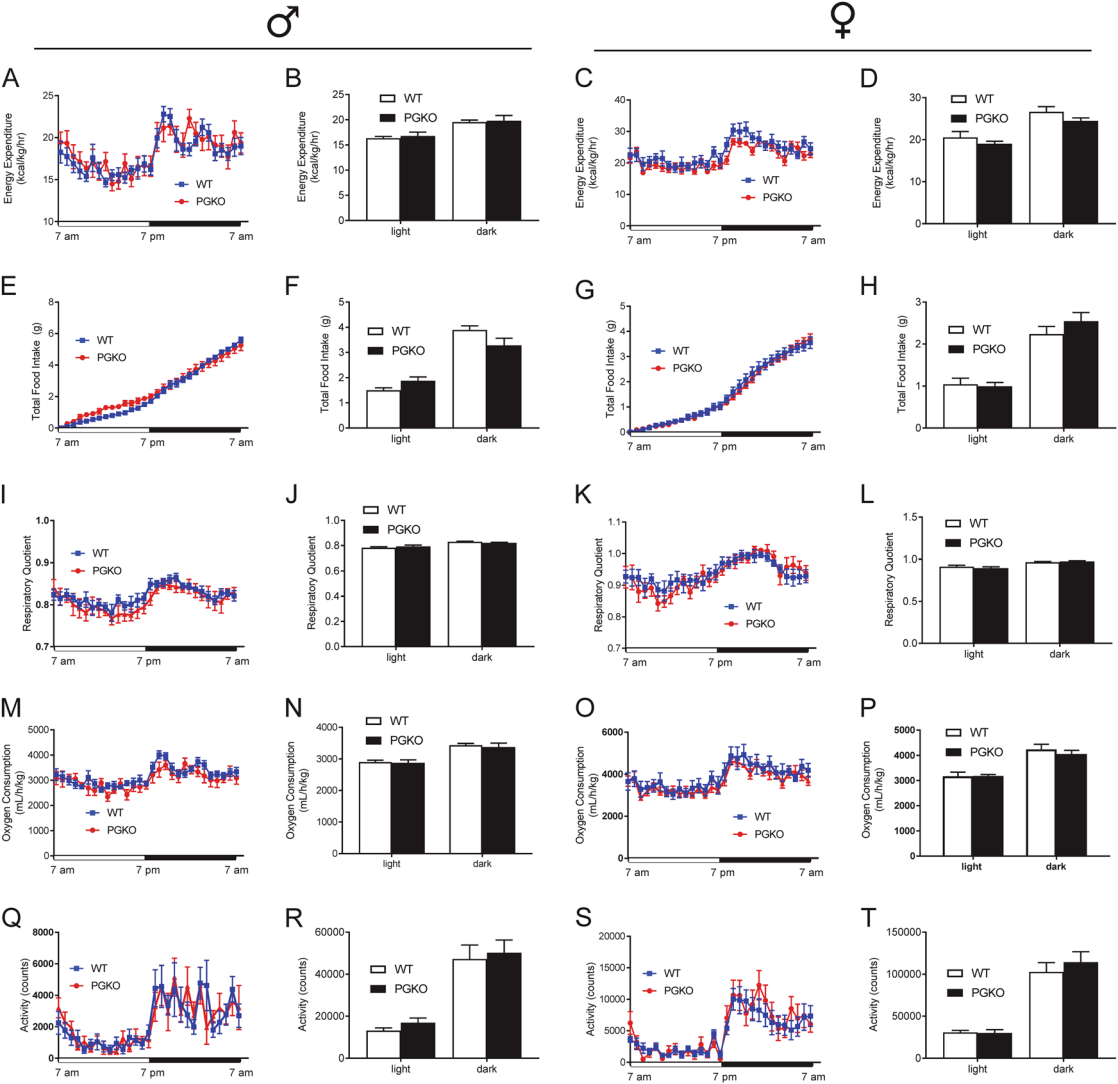
PGKO mice display comparable metabolic features as wild-type mice without dietary challenge. Left-hand figures are male mice; right-hand figures are female mice. We determined energy expenditure normalized to lean mass (**a**–**d**), total 24-h food intake (**e**–**h**), respiratory quotient (**i–l**), and oxygen consumption (**m**–**p**). Locomotor activity was measured by counting the number of floor beam breaks (**q**–**t**). Bar graphs are the 12-h average of data shown in corresponding line graph, separated into light and dark phases of the light cycle. Statistical comparisons with unpaired student’s *t*-test were non-significant (*n* = 8, 7 males; *n* = 9, 7 females). Data shown are average ± standard error

We used indirect calorimetry to determine whether male and female PGKO mice had differences in energy homeostasis on NCD. PGKO mice showed comparable measures compared with control mice in terms of energy expenditure (i.e., heat production, Fig. 2a–d), food intake (Fig. 2e–h), respiratory quotient (Fig. 2i–l), oxygen consumption (Fig. 2m–p), and locomotor activity (Fig. 2q–t). Since caloric intake and energy expenditure were similar between control and PGKO mice, mice did not develop different body weight/composition (Fig. 1a–d). The metabolism of glucose and fatty acids produces different respiratory quotients (volumetric CO_2_:O_2_ ratio) because of their different reaction stoichiometries. Respiratory quotient indicated that metabolic flexibility between carbohydrate or lipid metabolism was sustained for PGKO mice (Fig. 2m–p). Nocturnal locomotor activity (Fig. 1q–t) showed that PGKO mice had intact feeding behaviors in diurnal and nocturnal phases. Overall, PGKO mice did not have an overt metabolic phenotype when maintained on NCD.

### High-fat diet changes metabolism and feeding behaviors of PGKO mice in a sex-dependent manner

**Fig. 3:**
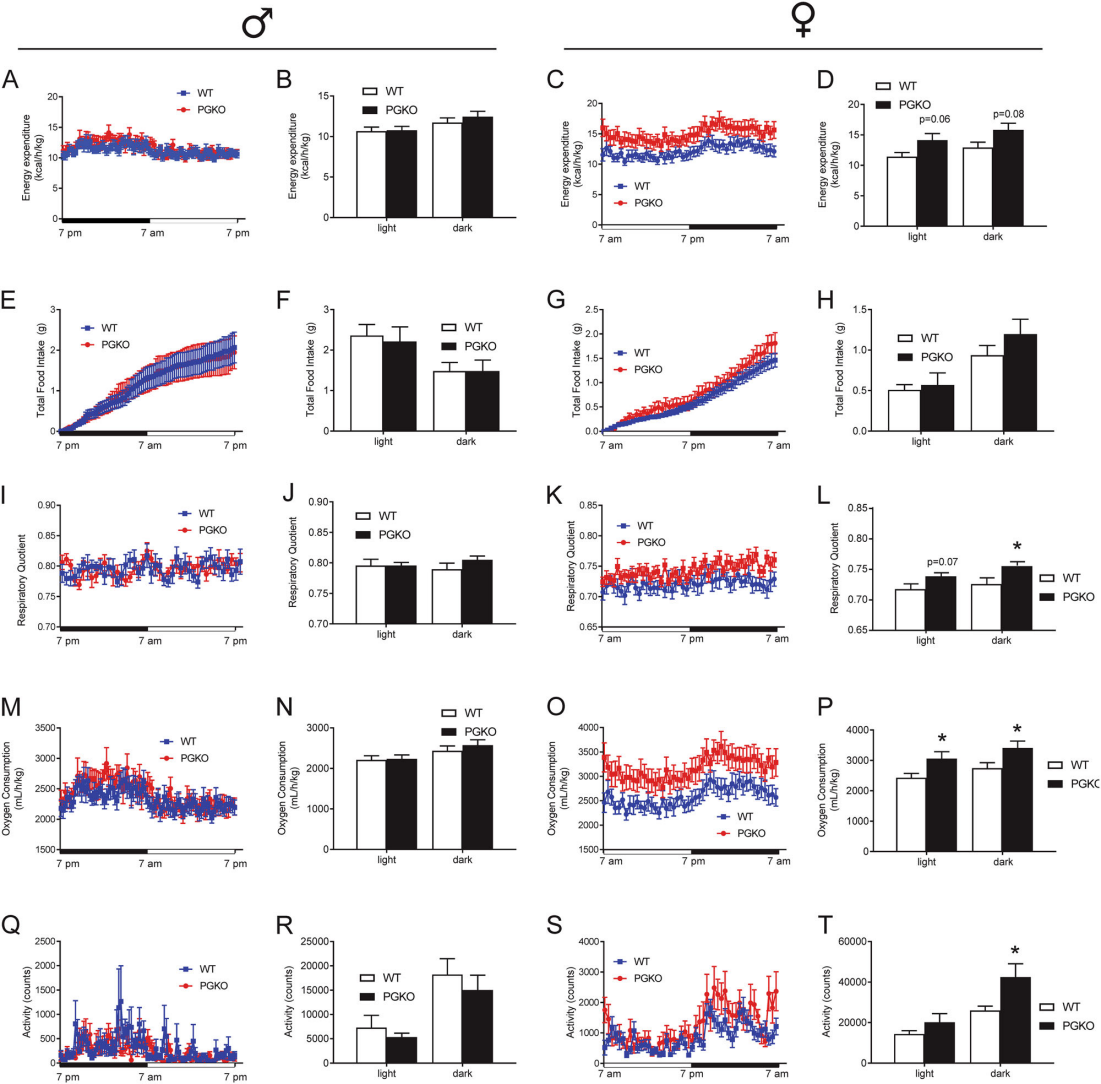
High-fat diet changes metabolism and feeding behaviors of PGKO mice in a sex-dependent manner. Mice were fed a long-term HFD ad libitum, then single-housed in metabolic caging for indirect calorimetry. Data shown are for ad libitum feeding during a 24-h window (12 h of light and dark as indicated on the *x*-axes). Left-hand figures are male data, right-hand figures are female data. We determined energy expenditure normalized to body weight (**a**–**d**), total 24-h food intake (**e**–**h**), respiratory quotient (**i**–**l**), and oxygen consumption (**m**–**p**). Locomotor activity was measured by counting the number of floor beam breaks (**q–t**). Bar graphs are the 12-h average of data shown in corresponding line graph, separated into light and dark phases of the light cycle. Data shown are average ± standard error. Unpaired Student’s *t*-test was used to compare WT and PGKO at the same light/dark phase (*n* = 8, 7 males; *n* = 10, 14 females). *p < 0.05 was considered significant; additional *p*-values are indicated for clarity

Since normal chow is a nutritionally balanced diet not sufficient to cause obesity, we decided to challenge PGKO mice with high-fat diet (HFD) containing 60% kcal from fat, 20% from protein, 20% from carbohydrate to reveal disparities in regulating energy homeostasis. One-year-old mice male and female mice were fed HFD for 3 and 5 months, respectively. We used indirect calorimetry to measure key metabolic features in both male and female mice and observed sexually dimorphic metabolic phenotypes. Male PGKO mice did not have statistically different energy expenditure during light or dark phases (Fig. 3a, b). By contrast, energy expenditure in female mice trended upward for light and dark phases but did not reach significance (Fig. 3c, d). Twenty-four hours of food intake was not significantly different in either the male or female groups (Fig. 3e–h). Together, these data suggested that female PGKO mice had better energy balance. Respiratory quotient was similar in male mice (Fig. 3i–j) but was significantly increased in female mice (Fig. 3k, l), indicating more glucose utilization in PGKO female mice. Oxygen consumption was not altered in male mice (Fig. 3m, n) but was significantly increased in female mice (Fig. 3o, p). We measured locomotor activity during the light and dark phase in PGKO mice (Fig. 3q–t) and found that female mice had significantly increased locomotor activity during the dark phase. Taken together, PGKO females appeared to have better energy balance and we predicted that these mice may resist weight gain with prolonged high diet feeding.

### PGKO mice resisted weight and adiposity gained from long-term HFD

**Fig. 4:**
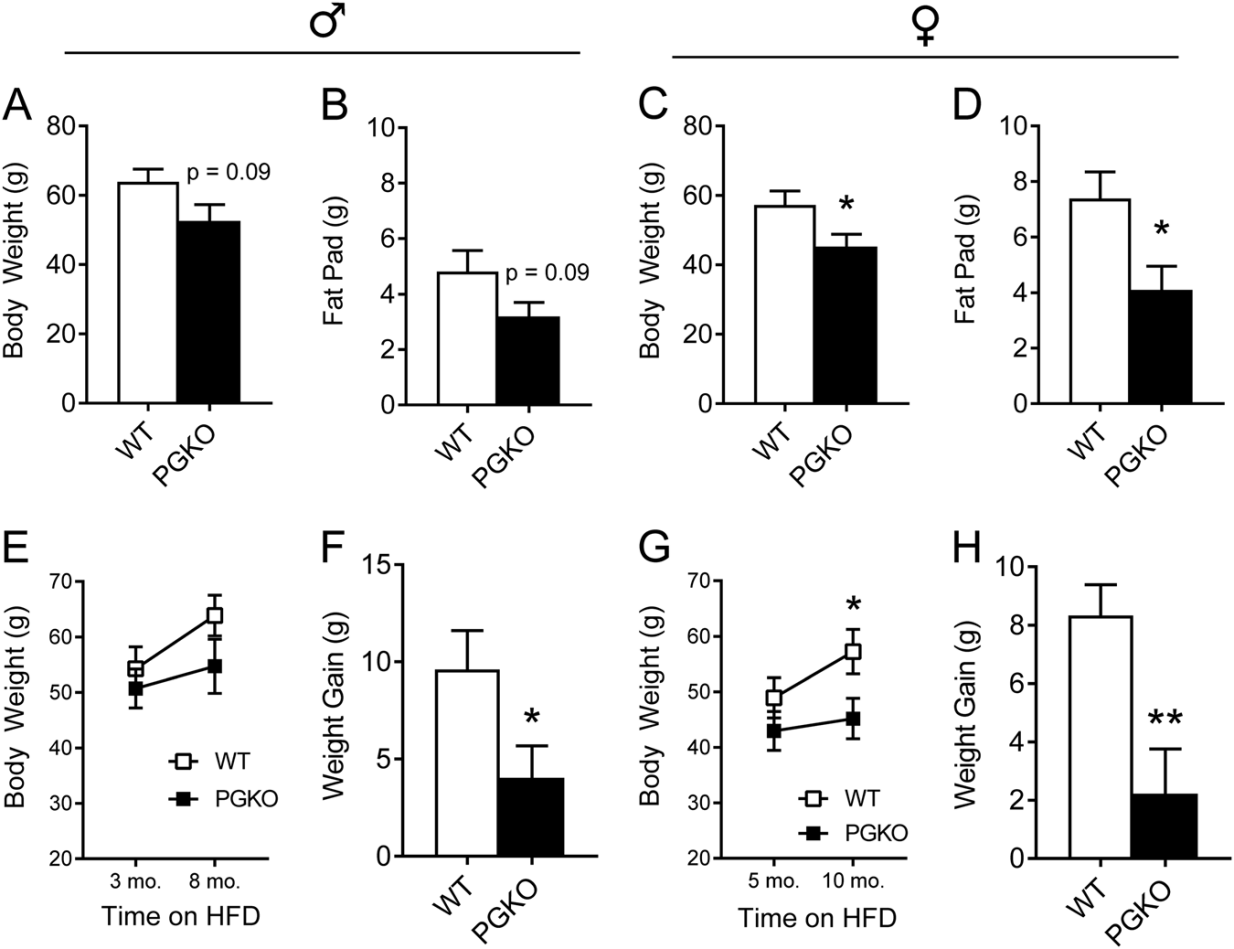
PGKO mice resisted weight and adiposity gained from long-term HFD. Left-hand figures are male data, right-hand figures are female data. Terminal body weight was measured in male (**a**) and female (**c**) mice after 8 and 10 months of HFD, respectively. Fat pad (i.e., epididymal white adipose tissue, EWAT) was dissected from euthanized mice and weighed for males (**b**) and females (**d**). Body weight was assessed at the time of indirect calorimetry and 5-months post calorimetry (see Fig. 3) for males (**e**) and females (**g**). The difference between these two time points (i.e., weight gain) was calculated for males (**f**) and females (**h**). Data shown are average ± standard error. Statistics were calculated with student’s *t*-test (*n* = 7, 8 male; *n* = 9, 11 female). *p < 0.05 was considered significant; **p < 0.01, additional *p*-values are indicated for clarity

In order to determine if improved energy balance predicted lower weight gain, we continued to feed HFD to the same cohorts used for the indirect calorimetry experiment (Fig. 3). Indeed, we discovered that after long-term HFD, male and female PGKO mice had lower total body weight (Fig. 4a, c, respectively) and epididymal white adipose tissue (EWAT) mass (Fig. 4b, d). Total body weight and EWAT mass trended lower in PGKO males but did not reach statistical significance (*p* = 0.09); however, weight gained from five additional months of HFD was significantly lower (Fig. 4f). The female PGKO group was even more protected from the diet-induced weight gain, having reduced body weight gain (Fig. 4g, h) and EWAT mass at the end of the experiment (Fig. 4d).

### PGKO mice have sex-dependent changes in serum metabolite concentrations and hepatic glycolytic enzyme expression

In order to determine if PGKO mice had altered nutrient metabolism compared with control mice, we measured the blood glucose and the serum concentration of various metabolites in other PGKO cohorts fed HFD. We determined that blood glucose levels during fasting-refeeding, glucose tolerance, and insulin tolerance were indistinguishable in PGKO mice (Fig. S2A–F). Then, we assessed whether the expression of key hepatic genes for glucose and lipid metabolism in PGKO mice was altered. We measured hepatic mRNA transcripts involved in glycolysis, gluconeogenesis, and fatty acid synthesis for males and females. In males, we found a trending decrease in glucokinase (*Gck*) and a significant twofold increase in pyruvate dehydrogenase kinase 4 (*Pdk4*) transcript (Fig. S2G). In female PGKO mice (Fig. S2H), we found a significant increase in glucokinase expression and trend for increased *Pdk4*. *Pck1* (encoding phosphoenylpyruvate carboxykinase) and *Pdk4* transcripts trended higher than control mice. Glucokinase is a target gene of insulin signaling through the sterol regulatory element binding transcription factor 1 (*Srebp1c*), which trended lower in PGKO males and females. When we profiled serum metabolite concentrations, we found male and female PGKO mice had sexually dimorphic alterations in several metabolites (Table 1). For male PGKO mice, triglyceride and glycerol concentration were increased during ad libitum feeding and short fast, respectively, indicating increased lipolysis. In female PGKO mice, there was a significant increase in non-esterified fatty acid during ad libitum feeding, and lower cholesterol during overnight fasting and refeeding. We concluded PGKO mice have increased peripheral metabolism of lipids and that hepatic enzymes were differently regulated to favor lipid utilization in PGKO mice, which could lead to the reduced adiposity gain after prolonged HFD feeding.

**Table 1:** PGKO mice have sexually dimorphic serum metabolite concentrations

	Ad libitum		Short fast		Overnight fast		Refeeding	
	Average ± SEM	*p*	Average ± SEM	*p*	Average ± SEM	*p*	Average ± SEM	*p*
*Male*								
Cholesterol (mg/dL)								
WT	24.74 ± 1.49	ns	17.37 ± 2.25	ns	21.48 ± 0.07	ns	24.70 ± 1.75	ns
PGKO	25.41 ± 1.78		19.92 ± 1.27		23.01 ± 0.11		24.75 ± 2.03	
Glycerol (mg/dL)								
WT	5.09 ± 0.32	ns	3.09 ± 0.27	0.01	2.52 ± 0.04	ns	4.61 ± 0.32	ns
PGKO	5.15 ± 0.29		3.98 ± 0.15		2.52 ± 0.05		4.76 ± 0.35	
Triglycerides (mg/dL)								
WT	95.1 ± 6.1	0.05	52.0 ± 3.4	ns	108.9 ± 7.4	ns	131.3 ± 8.7	ns
PGKO	117.6 ± 9.0		51.4 ± 2.5		96.6 ± 6.5		141.9 ± 7.3	
NEFA (mmol/L)								
WT	0.77 ± 0.06	ns	0.53 ± 0.05	ns	0.44 ± 0.02	ns	0.59 ± 0.04	ns
PGKO	0.76 ± 0.06		0.59 ± 0.04		0.40 ± 0.03		0.68 ± 0.05	
*Female*								
Cholesterol (mg/dL)								
WT	NA	NA	34.05 ± 1.69	ns	38.15 ± 2.11	0.01	39.00 ± 2.34	0.04
PGKO	NA		29.11 ± 2.56		27.85 ± 2.55		32.49 ± 1.60	
Glycerol (mg/dL)								
WT	5.01 ± 0.17	ns	3.96 ± 0.52	ns	4.30 ± 0.36	ns	5.32 ± 0.45	ns
PGKO	5.39 ± 0.56		3.66 ± 0.25		3.47 ± 0.71		5.05 ± 0.50	
Triglycerides (mg/dL)								
WT	65.4 ± 4.4	ns	54.1 ± 4.6	ns	84.5 ± 5.1	ns	111.5 ± 14.4	ns
PGKO	67.6 ± 5.6		49.8 ± 3.7		65.5 ± 12.5		113.1 ± 16.4	
NEFA (mmol/L)								
WT	0.51 ± 0.07	0.03	0.51 ± 0.03	ns	0.88 ± 0.04	ns	0.79 ± 0.05	ns
PGKO	0.80 ± 0.10		0.51 ± 0.06		0.69 ± 0.10		0.73 ± 0.10	

*NEFA* non-esterified free fatty acid, *NA* not assayed, *ns* not significant

Male data are shown in the upper half and female data are shown in lower half. Tail blood or cardiac blood were collected during various feeding regimens (see methods). Statistics were performed with unpaired students’ *t*-test, and *p* < 0.05 was considered significant (*n* = 11, 12 males; *n* = 9, 7 females)

### PGKO mice have sex-dependent increases in POMC processing

**Fig. 5:**
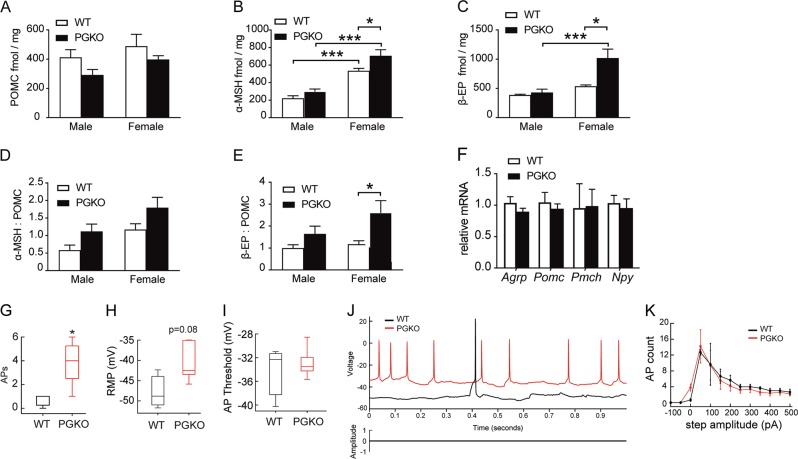
PGKO mice have increased POMC processing and POMC neuronal activity. **a**–**e** POMC-derived peptides from mediobasal hypothalamic blocks were measured in PGKO and control mice fed HFD for 2 weeks. POMC per mg total protein (**a**), α-MSH per mg total protein (**b**), and β-EP per mg total protein (**c**), α-MSH:POMC ratio (**d**), β-EP:POMC ratio (**e**), *n* = 4, 5 males; *n* = 4, 2 females. **f** Hypothalamic mRNA in female mice on HFD. **g**–**k** Electrophysiology recordings from fluorescently labeled POMC neurons from PGKO and control mice fed HFD for 2 weeks. **g** Number of spontaneous action potentials in POMC neurons. Mean RMP and AP threshold were measured in PGKO and control mice (**h**, **i**, respectively). **j** (top) Representative traces showing action potential (AP) firing and resting membrane potential (RMP) in POMC neurons. **j** (bottom) Protocol showing no current was injected when acquiring spontaneous AP recordings. **k** Number of action potentials evoked by stepwise increases in current amplitude. Electrophysiology recordings were collected from three WT and five KO neurons from male mice. Statistics were performed with two-way ANOVA and Sidak post hoc test (**a**–**e**) or student’s *t*-test (**g**–**k**); *p < 0.05 was considered significant; ***p < 0.001

POMC-derived neuropeptides play critical physiological functions, including appetite regulation^27^. α-MSH is anorexigenic, while the effect of β-endorphin (β-EP) on satiety is context-dependent^26,28,29^. The bioavailability of α-MSH and β-EP is, in part, determined by the expression of POMC and the subsequent proteolytic processing by PC1, PC2, and Cpe. We measured α-MSH, β-EP, and POMC neuropeptides in the mediobasal hypothalamic samples from mice fed HFD for 2 weeks. Prohormone POMC measurement was comparable between sexes and genotypes (Fig. 5a). PGKO mice, especially female knockouts, have increased POMC-derived α-MSH and β-EP (Fig. 5b, c). This led to greater α-MSH:POMC and β-EP:POMC ratios in PGKO mice (Fig. 5d–e). *Pomc* transcript as well as other appetite regulating peptides, *Agrp*, *Pmch*, and *Npy*, were similar in females (Fig. 5f). Since POMC transcripts and peptide were similar or trending downward, we conclude that the observed increases in α-MSH and β-EP are not a result of increased POMC synthesis and may result from effects on POMC processing. In addition, there is evidence that endogenous β-EP may complement the anorexigenic actions of α-MSH under certain conditions^28^. Taken together, we conclude that higher levels of hypothalamic α-MSH increases the anorexigenic tone in PGKO mice.

### Spontaneous action potential frequency is increased in POMC neurons from PGKO mice

Since increased electrical activity in POMC neurons suppresses appetite^5^, we used whole cell patch clamp recording in acute brain slices to measure spontaneous activity of POMC neurons in WT and PGKO mice. Gpr17 knockout neurons displayed a higher frequency of spontaneous action potentials (Fig. 5g, j) and depolarized resting membrane potential (*p* = 0.08) (Fig. 5h, j) at basal conditions, but similar AP threshold (Fig. 5i). Then, in order to determine the intrinsic capacity of POMC neurons to be excited by evoked current, we measured the frequency of evoked action potentials (APs) (Fig. 5k), which showed comparable results. We conclude that the increased spontaneous neuronal firing of POMC neurons contributes to the increased anorexigenic tone in PGKO mice.

It has been previously reported that a subset of Agrp/Npy neurons in the ARH express *Pomc* during development^30^, which could result in *Gpr17* deletion from a subset of Agrp/NPY neurons. To determine the relative number of AgRP/NPY neurons with *Gpr17* deletion, we used *Npy-Gfp* to label AgRP/NPY neurons and *Rosa-tdTomato* as a reporter for Pomc-Cre. We were able to identify that ~1/3 of labeled POMC neurons expressed *Npy-Gfp* (Fig. S3A, C). We found that AgRP/NPY neurons in PGKO mice had similar spontaneous action potential (sAP) and resting membrane potential (RMP, Fig. S3D, E). Transcripts encoding orexigenic and anorexigenic neuropeptides had no statistical differences (Fig. 5f). We concluded that *Gpr17* deletion in the subset of AgRP/NPY neurons does not change the orexigenic tone for PGKO mice on NCD.

### Leptin signaling in the arcuate cells of PGKO mice

Leptin increases the excitability of POMC neurons and thus promotes satiety^31^. Therefore, we determined if altered leptin signaling may explain the improved energy balance in Gpr17-deficient mice. In order to determine if PGKO mice had altered sensitivity to leptin in the arcuate nucleus, we used immunohistochemistry to quantify leptin signaling by measuring downstream Stat3 phosphorylation (pStat3). After refeeding, the number of pStat3 + cells (Fig. S4A, B) and the intracellular pStat3 intensity (Figure S4 C) were modestly increased in PGKO sections, suggesting that PGKO mice have normal sensitivity to leptin on NCD. In HFD-fed mice, the concentration of circulating leptin was unchanged after refeeding (Fig. S4D, E), which suggests that PGKO mice retain normal sensitivity to leptin signaling in the arcuate nucleus.

## Discussion

Our POMC neuron-specific Gpr17 knockout model reveals a physiological role for Gpr17 signaling in regulating energy homeostasis through POMC neurons. PGKO mice were protected from diet-induced obesity, which resulted in lower total body weight and lower EWAT mass for both males and females. Interestingly, female PGKO mice were more protected from long-term obesity than males. We used functional assays of POMC neurons to determine the cellular mechanisms underlying the metabolic phenotypes. We observed sex-dependent increases in POMC-derived peptides (α-MSH and β-EP) from hypothalamic extracts collected after 2 weeks of HFD exposure in PGKO mice. Our electrophysiological results showed that the spontaneous firing of Gpr17-deficient POMC neurons was increased. We concluded that Gpr17 deletion increased the POMC neuronal activity and promoted better energy homeostasis that curtailed weight gain, especially in female mice. Since male PGKO mice had only partial increases in POMC-derived peptide production compared with female PGKO mice, they were less protected from weight gain.

Sexual dimorphism is well documented for metabolic regulation. Estrogen signaling has been reported as the underlying cause for the gender-specific modulation of abdominal adiposity^32^, adipose tissue lipolysis^33^, and hepatic glucose metabolism^34^. The gender-specific difference in metabolism is more evident with HFD feeding in the adipose and brain tissues^34–36^. Of note, the quantity and physiology of hypothalamic POMC neurons are sex-dependent. In females, POMC neurons are more numerous, have increased electrical activity, and elevated hypothalamic expression of POMC transcript and protein^23,37^. Biological mechanisms regulating POMC neurons are also sex-specific. Leptin signaling in POMC neurons (which increases POMC neuronal firing) also has sex-dependent effects on adipose acquisition, energy expenditure, peripheral glucose tolerance, and locomotor activity^38^. Moreover, estrogen modulates food intake through receptors expressed by POMC neurons^39^. Elucidating how targeting specific signaling pathway in hypothalamic neurons to improve energy homeostasis in a gender-specific manner warrants future investigation.

Hypothalamic neurocircuits, including POMC neurons, have been shown to increase autonomic outflow to peripheral organs, which increases lipolysis in adipose tissues and insulin release by pancreatic islets^40–43^. Our approach for conditional deletion of *Gpr17* in POMC neurons resulted in alterations in liver mRNAs related to glucose utilization. This data is consistent with previous findings that connect POMC neuronal activity with hepatic glucose production and insulin sensitivity. For example, chemogenetic stimulation of POMC neurons rapidly induced sympathetic nerve activity to the liver^44^. Central administration of α-MSH increases hepatic gluconeogenesis and the requisite enzymes Pepck and G6pase^45^. The vagal nerve, which connects brain and liver, suppresses hepatic glucose production^46^. Moreover, insulin action in the CNS alters hepatic glucose production and requires vagal efferents^46^. Therefore, liver mRNAs that are upregulated in PGKO mice could be a direct consequence of altered POMC neuronal activity.

It is also important to note that several age-related mechanisms impair POMC neuronal function and contribute to obesity. Gpr17 knockout may restore some of these pathways that are eroded by aging^19^. For example, as mice age, POMC neurons are electrically silenced by elevated activation of mTOR and hyperactive K_ATP_ channels^47^. HFD-fed obese mice have severe leptin resistance in POMC neurons^48^. The synaptic input from AgRP/NPY neurons to POMC neurons increases^47,48^, which progressively increases orexigenic tone in older mice^49^. In our study, PGKO mice, especially females, were protected from adiposity gained due to age and HFD feeding. Although males did not have an apparent change in energy homeostasis, female PGKO mice had increased energy expenditure, nighttime locomotor activity, and increased glucose utilization. It is possible that deficiency in Gpr17 signaling protects against conditions that silence POMC neurons, such as HFD and age.

Our group published previously that Gpr17 substantially altered energy homeostasis in AgRP neuron-specific Gpr17 knockout mice^20^. Since AgRP and POMC neurons have opposing actions on energy homeostasis, one of our principle research questions was whether Gpr17 activity in POMC neurons counteracted that of AgRP neurons. In this regard, PGKO mice surprisingly also had a favorable effect on obesity. One possible explanation is that AgRP conditional knockout mice had enhanced leptin sensitivity of ARH neurons, which was also the trend in PGKO mice. Since leptin signaling oppositely regulates AgRP and POMC neuronal activity, both mice may have favorable metabolic profiles due to sensitization to leptin. The fact that Gpr17 knockout produced positive, but unique, metabolic outcomes in *Agrp* and *Pomc* conditional knockout mice supports its potential role as a target for obesity therapy.

In addition, Gpr17 expression is not restricted to POMC and AgRP neurons. Other groups have identified its roles in myelination and metabolic role in oligodendrocytes^50^, mitigating ageing-related defects and ischemic damage in the brain^19,51^ and heart^52^. The inconsistent report on whole-body Gpr17 knockout^50,53^ is probably due to the physiological compensation and developmental adaption that are often associated with germ-line knockouts, which limits the interpretation of negative data as a true absence of a function^54^. Therefore, our studies using conditional knockout mice provide insights on the metabolic function of this orphan GPCR. Future studies with conditional knockout in peripheral tissues are warranted in order to understand the integrative physiology of Gpr17.

In conclusion, our results suggest a role for Gpr17 in regulating energy homeostasis and metabolism through POMC neurons. Thus, eliminating the endogenous agonist or supplying antagonists of Gpr17 could be therapeutically relevant strategies for managing obesity.

## Supplementary information

Figure S1

Figure S2

Figure S3

Figure S4

Supplemental Figure Legends

Supplemental Methods
