# Supplementary material for: Gpr17 deficiency in POMC neurons ameliorates the metabolic derangements caused by long-term high-fat diet feeding
Source: Nutr Diabetes. 2019 Oct 14;9:29. doi: 10.1038/s41387-019-0096-7 (PMC6791877; doi:10.1038/s41387-019-0096-7)
Supplement: Supplementary file 5 — Supplemental Figure Legends [file 41387_2019_96_MOESM5_ESM.docx]

**Supplemental figure legend**

**Figure S1.** PGKO mice have selective Gpr17 ablation in POMC neurons. A) Genomic DNA was extracted from various tissues of a PGKO mouse. Recombination band (Δ, 300 bp) and lox band (200 bp) are indicated. Arrow indicates a faint Δ band in the MBH sample. Pomc-Cre was detected in all tissues (not shown). B-E) mRNA was collected from POMC neurons that were sorted based on Tomato expression (Tomato+) and compared to input (i.e. unsorted) cells. Amplification of *Gpr17* and *β-Actin* (*Actb)* was confirmed by gel electrophoresis (B). Gpr17 mRNA from Tomato+ cells (C) was detected in WT, but not detected in PGKO mice. Gpr17 transcript in input cells (D) was reduced in PGKO mice. POMC transcript (E) was enriched ~400-fold by Tomato+ cell sorting. mbh: mediobasal hypothalamus; bs: brainstem; cx: cerebral cortex; liv: liver; int: small intestine; sp: spleen; sm: skeletal muscle; wa: white adipose; c: water control for PCR; N.D.: not detectable.

**Figure S2.** PGKO mice have sex-dependent changes in hepatic glycolytic enzyme expression. Left-hand figures are male data, right-hand figures are female data. Tail blood glucose was evaluated during various feeding regimens (A-B). Insulin action was determined with glucose tolerance tests (C-D) and insulin tolerance tests (E-F). Liver mRNA expression was measured in refeeding male mice (G) and *ad libitum*-fed female mice (H) on HFD. Data shown are average ± standard error. Statistics were calculated with unpaired student’s t-test (n=11,12 males; n= 9,7 females). p < 0.05 (*) was considered significant.

**Figure S3.** PGKO mice have normal electrical activity in AgRP/NPY neurons. A subpopulation of AgRP/Npy neurons activate Cre during development. A) Coronal cross section of the arcuate nucleus of the hypothalamus. Red: Pomc-Cre irreversibly activates Tomato expression after Cre-mediated recombination. Green: Npy-Gfp expression, which is restricted to AgRP neurons in the ARH. Yellow: Overlap of red and green signal. B) Zoom-in image of the box in (A). C) Venn Diagram of average number ± standard error of neurons that are Tomato positive-only (red), Npy-gfp positive-only (green), or double positive (yellow) in the ARH (n= 4 mice). Scale bars are 100 μm. Spontaneous action potential (sAP) frequency (D) and resting membrane potential (RMP) (E) were determined by whole cell patch clamp of Npy-GFP neurons in WT and PGKO brain sections (n=20-23). Statistical comparisons with unpaired student’s t-test were non-significant.

**Figure S4.** Leptin signaling intermediate phospho-Stat3 is unaltered in arcuate cells of PGKO mice on NCD. Brains were collected from PGKO and control male mice raised on NCD after refeeding. A) 10 μm thick coronal brain sections were stained with pStat3 antibody and DAPI in control and PGKO male mice. Scale bars shown are 50 μm. B) Average number of pStat3+ cells in the ARH (n=2 per group). C) Histogram of intracellular pStat3 intensities for WT and PGKO brain sections. D-E) Serum leptin concentration in HFD-fed male (D) and female (E) mice during refeeding.
