# Supplementary material for: Gpr17 deficiency in POMC neurons ameliorates the metabolic derangements caused by long-term high-fat diet feeding
Source: Nutr Diabetes. 2019 Oct 14;9:29. doi: 10.1038/s41387-019-0096-7 (PMC6791877; doi:10.1038/s41387-019-0096-7)
Supplement: Supplementary file 6 — Supplemental Methods [file 41387_2019_96_MOESM6_ESM.docx]

**Supplemental Methods**

**Histology**. We processed mouse brains as described previously (17). In brief, mice were transcardially perfused with 4% paraformaldehyde before dissection. Brains were fixed in 4% paraformaldehyde overnight, then after sucrose saturation, brains were frozen in OCT compound. 10 μm-thick coronal sections containing the ARH (bregma position approximately -1.50 mm) were collected. For imaging Npy-Gfp and Tomato, sections were stained with DAPI (Sigma) and imaged with a confocal microscope (Leica LSM 700). We counted the number of DAPI/Gfp/Tomato positive neurons in the ARH with NIH ImageJ software. For imaging pStat3 immunohistochemistry, sections were stained with Phospho-Stat3 (Tyr705) rabbit anti-mouse antibodies (Cell Signaling 9131) and Alexa Fluor 555 conjugated goat anti-rabbit secondary antibodies (Invitrogen) followed by DAPI staining. Intracellular pStat3 signal intensity for all cells in the ARH was quantified with NIH Image J software. For counting pStat3 positive (pStat3+) cells, all images were processed with a common lower threshold and counted based on contiguous signal and morphology.

**POMC neuron electrophysiology.** Coronal brain slices (300 µM) containing the hypothalamus were prepared from male Gt(Rosa)26Sor^tm9(CAG-tdTomato)Hze^ (Jax #007914) transgenic WT and PGKO mice (8-10 weeks old). Cre-mediated recombination labels cells with tdTomato expression. Mice were briefly (~15 seconds) anesthetized with 99.9% isoflurane (Patterson Veterinary), and decapitated. Brains were dissected quickly and chilled in ice-cold, oxygenated (95% O2, 5%CO2) section solution (in mM: 110 choline chloride, 25 NaHCO3, 25 D-glucose, 11.6 sodium ascorbate, 7 MgSO4, 3.1 sodium pyruvate, 2.5 KCL, 1.25 NaH2PO4, and 0.5 CaCl2) using a Leica VT1200S vibratome. Prior to recordings, slices were maintained for 30 min at 37°C in oxygenated (95% O_2_, 5% CO_2_) artificial CSF (aCSF) bath (in mM: 127 NaCl, 25 NaHCO3, 25 D-glucose, 2.5 KCl, 1MgCl2, 2 CaCl2, and NaH2PO4). Slices were subsequently incubated for at least 45 min in aCSF at room temperature before recording. Whole-cell patch recordings were performed using borosilicate glass electrodes (3-4 MΩ resistance) filled with an intracellular solution (in mM: 128 K-gluconate, 10 HEPES, 1 EGTA, 4 MgCl2, 4 ATP, 0.4 GTP, 10 phosphocreatine, 3 ascorbate, and 0.05 Alexa Fluor 488 hydrazide (Invitrogen) plus 4 mg/ml biocytin (Sigma-Aldrich)). Slices were placed in the recording chamber of a SliceScope Pro 6000 (Scientifica) and continuously perfused with oxygenated aCSF at the rate of ~1 mL per minute at 30-31°C. Slices were held in place with harp (Warner). POMC neurons were identified by fluorescence with a LED illumination system (CoolLED pE-4000) and patched under IR-DIC optics. Recordings were acquired with a Multiclamp 700B amplifier (Molecular Devices), amplified and filtered at 4 kHz and digitized at 10 kHz. Membrane potential was held at -70mV in a voltage-clamp mode. Pipette capacitance was compensated and inclusion of data required a series resistance < 35 MΩ. Current-clamp recordings were bridge balanced.

**NPY neuron electrophysiology.** Coronal brain slices (300 um) containing the hypothalamus were prepared from female Npy-Gfp (Jax #006417) transgenic WT and PGKO mice (8-10 weeks old). Mice were sacrificed by decapitation, brains were rapidly removed and slices were cut in ice-cold, oxygenated (95% O2, 5%CO2) section solution (in mM: 10 NaCl, 25 NaHCO3, 2.5 KCl, 10 D-glucose, 1.25 NaH2PO4, 195 sucrose, 2 NaN3, 7 MgCl2, 1 CaCl2, pH 7.3) using a Leica VT1200 vibratome. Prior to recordings, brain slices were maintained at room temperature for at least 1 h in oxygenated (95% O2, 5% CO2) artificial cerebrospinal fluid (aCSF) (in mM: 120 NaCl, 5 KCl, 26 NaHCO3, 1.2 NaH2PO4, 2CaCl2, 1 MgCl2, 5 D-glucose, pH 7.4). Mannitol was used to adjust the osmolality of the low glucose aCSF (0.1 mM). Whole-cell patch recordings were performed using borosilicate glass electrodes with resistance (4-6 MΩ resistance) were filled with an intracellular solution (in mM: 128 K-gluconate, 10 KCl, 10 Hepes, 0.1 EGTA, 2 MgCl2, 3 ATP and 0.1 GTP, pH 7.3). Npy-Gfp neurons in the ARH were identified by fluorescence using a LED illumination system (CoolLED pE-4000) and patched under IR-DIC optics. Recordings were acquired with an Axopatch 700B amplifier, digitized and analyzed with pClamp10 software (Molecular Devices).

**Electrophysiology data analysis and statistics**. Offline data analysis was performed using Custom MATLAB (The MathWorks, RRID:SCR_001622). For the intrinsic data comparison, parametric unpaired t-test was used for normally distributed data, while nonparametric unpaired t-test was used for non-normally distributed data. Differences were considered significant a p < 0.05. Unless otherwise noted, results are presented as mean ± SEM. For data presented as boxplots, the box displays the central 50% of the data with the central line indicating the median and the lower/upper boundary lines being the 25%/75% quantile of the data.

**Flow Assisted Cell Sorting (FACS).** Neurons were collected from the mediobasal hypothalamus of PGKO or control mice on postnatal day 11-13 using a papain dissociation kit (Worthington Biochemicals). Neurons were pooled by genotype from 4-5 mice of both sexes. Live neurons were gated for Tomato-expressing cells and compared with input (i.e. unsorted) cells for downstream gene expression analysis.
